# Standardization of Epidemiological Surveillance of Acute Rheumatic Fever^[Author-notes ofac252-FM1]^

**DOI:** 10.1093/ofid/ofac252

**Published:** 2022-09-15

**Authors:** Amy Scheel, Andrea Z Beaton, Judith Katzenellenbogen, Tom Parks, Kate M Miller, Thomas Cherian, Chris A Van Beneden, Jeffrey W Cannon, Hannah C Moore, Asha C Bowen, Jonathan R Carapetis

**Affiliations:** Emory University School of Medicine, Atlanta, Georgia, USA; Cincinnati Children’s Hospital Medical Center, The Heart Institute, Cincinnati, Ohio, USA; School of Population and Global Health, University of Western Australia, Perth, Australia; Department of Infectious Disease, Imperial College London, Hammersmith Hospital; Wesfarmers Centre of Vaccines and Telethon Kids Institute, The University of Western Australia, Nedlands, Western Australia; MMGH Consulting, Geneva, Switzerland; CDC Foundation, Atlanta, Georgia, USA; Wesfarmers Centre of Vaccines and Telethon Kids Institute, The University of Western Australia, Nedlands, Western Australia; Department of Global Health and Population, Harvard T.H. Chan School of Public Health, Boston, Massachusetts, USA; Wesfarmers Centre of Vaccines and Telethon Kids Institute, The University of Western Australia, Nedlands, Western Australia; Wesfarmers Centre of Vaccines and Telethon Kids Institute, The University of Western Australia, Nedlands, Western Australia; Perth Children’s Hospital, Nedlands, Western Australia; Wesfarmers Centre of Vaccines and Telethon Kids Institute, The University of Western Australia, Nedlands, Western Australia; Perth Children’s Hospital, Nedlands, Western Australia

**Keywords:** rheumatic fever, *Streptococcus*, surveillance

## Abstract

Acute rheumatic fever (ARF) is a multiorgan inflammatory disorder that results from the body’s autoimmune response to pharyngitis or a skin infection caused by *Streptococcus pyogenes* (Strep A). Acute rheumatic fever mainly affects those in low- and middle-income nations, as well as in indigenous populations in wealthy nations, where initial Strep A infections may go undetected. A single episode of ARF puts a person at increased risk of developing long-term cardiac damage known as rheumatic heart disease. We present case definitions for both definite and possible ARF, including initial and recurrent episodes, according to the 2015 Jones Criteria, and we discuss current tests available to aid in the diagnosis.

We outline the considerations specific to ARF surveillance methodology, including discussion on where and how to conduct active or passive surveillance (eg, early childhood centers/schools, households, primary healthcare, administrative database review), participant eligibility, and the surveillance population. Additional considerations for ARF surveillance, including implications for secondary prophylaxis and follow-up, ARF registers, community engagement, and the impact of surveillance, are addressed. Finally, the core elements of case report forms for ARF, monitoring and audit requirements, quality control and assurance, and the ethics of conducting surveillance are discussed.

## Teaser

This article presents case definitions for acute rheumatic fever and best practice protocols for conducting epidemiological surveillance for acute rheumatic fever in a variety of settings.

## DISEASE CHARACTERISTICS

Acute rheumatic fever (ARF) is a nonsuppurative, delayed sequela of a *Streptococcus pyogenes* (Strep A) infection through an inflammatory response in untreated or undertreated susceptible individuals. Symptoms of ARF typically begin 3 weeks (range: 1 to 5) following the acute Strep A infection. At least 470 000 cases of ARF occur every year worldwide [1]. The incidence of ARF ranges from 8 to 51 per 100 000 children and young adults worldwide, with reports as high as 200 per 100 000 in endemic areas [2, 3]. However, the true incidence of ARF is hard to assess given that it occurs mostly in places such as Asia and Africa, where robust regional and centralized data are sparse.

Acute rheumatic fever usually affects children aged 5 to 15 years, because pharyngeal and skin infections with Strep A are common in this population. Untreated Strep A infections carry a 3% risk of ARF; however, the risk of recurrences rises to more than 50% in children in young adults who have had a previous ARF episode [4, 5].

Acute rheumatic fever is a multiorgan inflammatory disorder affecting the heart (carditis), joints (arthritis and arthralgia), brain (Sydenham’s chorea), skin (erythema marginatum), and subcutaneous tissue (subcutaneous nodules) [6]. Although episodes of ARF can result in significant short-term disability, the major impact of ARF at the population level is that it causes long-term, irreversible damage to heart valves—termed rheumatic heart disease (RHD)—often as a result of recurrences [7]. Diagnosis of ARF is important, because antibiotic prophylaxis can prevent progressive valvular heart disease and the development of cardiovascular complications [8]. Primary prevention of RHD involves the identification and treatment of Strep A infections. However, only 60% of patients who develop ARF have a history of clinical pharyngitis. Thus, identifying patients with ARF is another opportunity for early intervention with secondary prevention through antibiotic prophylaxis. Most patients with a history of ARF and mild RHD who avoid recurrent episodes of ARF will have no detectable cardiac disease in 5–10 years [9, 10].

Improving the global burden estimate through disease surveillance is critical for characterizing the epidemiology of ARF and providing essential information on disease progression to inform future vaccine implementation strategies. The establishment of surveillance sites has the further benefit of providing a means for evaluating interventions such as vaccines and therapies.

## OBJECTIVES OF SURVEILLANCE FOR ACUTE RHEUMATIC FEVER

An effective surveillance system for ARF serves to (1) determine the age- and sex-specific incidence (preferred) or period prevalence of initial, recurrent, and possible ARF cases; (2) monitor trends in demographic and clinical characteristics of persons diagnosed with ARF; and (3) monitor outcomes, including the development of RHD in persons with a history or confirmed or suspected ARF.

### Secondary Objectives

Surveillance systems may also aim to determine the antecedent infection for initial and recurrent ARF cases; for example, pharyngitis or skin infection.

## CASE DEFINITIONS AND DIAGNOSTIC CRITERIA

Standardized case definitions are important for obtaining accurate surveillance data, enabling comparisons of surveillance data across jurisdictions, and monitoring the impact of interventions. The definitions and methods presented here may also be used as clinical endpoints for vaccine efficacy trials and for postlicensure effectiveness studies. Case definitions for ARF are based on the Jones Criteria 2015 revision (Table 1), with some modifications accounting for recent advances (Supplementary Appendix 1). Definite and possible case definitions for ARF are shown in Table 2. The Jones Criteria were developed by an international advisory group of experts organized by the American Heart Association’s Council on Cardiovascular Disease in the Young and its Rheumatic Fever, Endocarditis, and Kawasaki Disease Committee [11].

**Table 1.** 2015 Revised Jones Criteria for the diagnosis of ARF [11]

Moderate and high-risk populations	Low-risk populations^a^
**Major Criteria**	
Carditis: clinical and/or subclinical^b^	Carditis: clinical and/or subclinical^b^
Arthritis: monoarthritis or polyarthritis	Arthritis: polyarthritis only
Polyarthralgia^c^	
Chorea	Chorea
Erythema marginatum	Erythema marginatum
Subcutaneous nodules	Subcutaneous nodules
**Minor Criteria**	
Monoarthralgia	Polyarthralgia
Fever >38.0°C	Fever >38.5°C
ESR ≥30 mm/hour and/or CRP ≥3.0 mg/dL^d^	ESR ≥60 mm in the first hour and/or CRP ≥3.0 mg/dL^d^
Prolonged PR interval, after accounting for age variability (unless carditis is a major criterion)	Prolonged PR interval, after accounting for age variability (unless carditis is a major criterion)

Abbreviations: ARF, acute rheumatic fever; CRP, C-reactive protein; ESR, erythrocyte sedimentation rate.

NOTE: Additional guidance on using the Revised Jones Criteria can be found in Supplementary Appendix 1.

a A population is considered low risk if the incidence of ARF is < or = to 2 per 100,000 school-aged children or all-age RHD prevalence of < or = to 1 per 1000 population per year.

b See Supplementary Appendix 1.

c See Supplementary Appendix 1.

d See Supplementary Appendix 1.

**Table 2.** Case Definitions for ARF^a^

**Definite ARF**
***Initial ARF:*** The presence of 2 major manifestations OR 1 major and 2 minor manifestations of ARF as defined by the 2015 Revision of the Jones Criteria, with evidence of preceding, laboratory-confirmed, Strep A infection [11]*, in a person with no previous history of ARF or RHD.
*Exceptions: Chorea and indolent carditis are considered stand-alone criteria for establishing an ARF diagnosis and do not require evidence of preceding Strep A infection.
***Recurrent ARF:*** The presence of 2 major manifestations, or 1 major and 2 minor manifestations, or 3 minor manifestations of ARF as defined by the Jones Criteria (2015 revision), with evidence of a preceding, laboratory-confirmed, Strep A infection [11], in a person with a reliable history of ARF or RHD, with at least 90 days from onset of last episode of ARF.
**Possible ARF**
***Possible initial ARF:*** A possible ARF case is defined as illness in a person who fulfills the criteria for ARF—(a) 2 major criteria or (b) 1 major and 2 minor criteria—but does not have evidence of prior Strep A infection on laboratory testing, does not have access to such testing or has a presumed or confirmed strep A skin infection, or (c) presents with 1 major and 1 minor criteria WITH evidence of streptococcal infection and no confirmable alternate diagnosis for the presenting symptoms.
***Possible recurrent ARF:*** A possible recurrent ARF case is defined as illness in a person with a reliable history of ARF or RHD with at least 90 days from onset of last episode, who fulfills the criteria for ARF—(a) 2 major criteria or (b) one major and 2 minor criteria—but does not have evidence of prior Strep A infection on laboratory testing or does not have access to such testing, has a presumed or confirmed strep A skin infection, or (c) presents with 1 major and 1 minor criteria WITH evidence of streptococcal infection and no confirmable alternate diagnosis for the presenting symptoms.
^*^ *Note about Possible ARF:* The Jones Criteria (2015 revision) provides the option of “possible” ARF, where there is strong clinical suspicion of ARF, but diagnostic criteria are not met. In some national guidelines (eg, Australia), this is called “probable” ARF with a further diagnostic category of possible ARF denoting those in which there is greater diagnostic uncertainty, which can create some confusion. We have used the Jones Criteria definitions because they can be universally used. However, we recommend clearly defining which terms will be used before surveillance so comparison between countries is feasible. Finally, although not currently included in the Jones Criteria as an indicator of Strep A infection, recent research suggests that Strep A skin infections are a likely cause of ARF [12]. As evidence continues to emerge, we think it is critical to contribute to this data gap and collect surveillance data for all persons presenting with ARF secondary to impetigo. We recommend that all cases of ARF in which the only confirmed laboratory evidence of Strep A infection is a skin culture be called “Possible ARF”. If a person meets full criteria, including one of the other measures indicating prior or current Strep A infection, included in the Jones Criteria, such as elevated antistreptolysin (ASO) or anti-DNase B (ADB), which is often seen with Strep A skin infections, then definite ARF should be used.

Abbreviations: ARF, acute rheumatic fever; RHD, rheumatic heart disease; Strep A, *Streptococcus pyogenes*.

## MICROBIOLOGICAL, LABORATORY, AND CLINICAL TESTS USED IN THE DIAGNOSIS OF ACUTE RHEUMATIC FEVER

Laboratory confirmation of a preceding Strep A infection for the diagnosis of ARF can be achieved by 3 different means: throat culture, nucleic acid amplification tests (NAAT) or rapid antigen detection testing (RADT), or streptococcal serology. Exceptions to this include chorea and indolent carditis, which may only become apparent after the precipitating Strep A infection, by which time antibody levels may no longer be elevated. Laboratory testing for antistreptolysin (ASO) or anti-DNase B (ADB) titers is the most useful method to determine recent infection with Strep A in ARF. A summary of the key features, advantages, and disadvantages of these diagnostic methods are in Supplementary Appendix 2.

### Antistreptolysin and Anti-DNase B Titers

It is recommended that acute serum be collected as soon as possible after the onset of ARF symptoms, and that the antibody titer be compared with a convalescent sample to detect a rise in titer. The ASO test is usually obtained first; if it is not elevated, an ADB test may be performed. A 4-fold increase in titer from acute to convalescent (taken at least 2 weeks apart, preferably 4-6 weeks apart) is considered gold standard, however a two fold increase is considered acceptable [11, 13].

An upper limit of normal (ULN) cutoff (80th percentile) can be used when paired acute and covalescent titers are not available. Because symptoms of ARF typically do not appear until several weeks after the acute Strep A infection, by which time ASO and ADB are likely elevated, ULN may be the best test of antecedent Strep A infection. Age-stratified ULN values for serum ASO and ADB titers for a subset of healthy individuals (without recent streptococcal infection) from the surveillance population of interest should be used if already available as threshold titers because values can differ between and within countries based on variables such as ethnicity, geography, and socioeconomic status [14]. Given the logistic and cost burden associated with establishing population titers, we recommend using population values if available, but it is not required to obtain in the population of interest if not readily available. See Supplementary Appendix 3 for ASO and ADB ULN titers when local derivations are not available.

### Bacterial Culture

Because it takes 2–3 weeks for the immune system to produce antistreptococcal antibodies, serology testing is often negative during the acute Strep A infection; culture and RADT are often more useful in this scenario. Culture is additionally necessary to obtain the Strep A isolate if further characterization such as *emm* typing antibiotic susceptibility testing, or whole-genome sequencing, is part of the surveillance program.

Culture of swabs is performed in a laboratory setting. Clinical throat swabs are typically inoculated onto blood agar plates; however, selective plates can be used [15]. Inoculated agar plates are initially incubated at 37°C for 18–24 hours, but incubation up to 48 hours may be necessary. The addition of 5%–10% CO_2_ for incubation may enhance growth but is not essential. After incubation, plates are inspected for β-hemolytic colonies to undergo subculture purification and confirmation with further biochemical tests including latex agglutination testing for Lancefield groups A, C, G, bacitracin sensitivity and pyrrolidonyl arylamidase testing. No biochemical test is 100% specific for *S. pyogenes* and are therefore frequently performed in combination [16]. Purified colonies can be stored enabling further testing, with long-term storage between −70 and −80°C in a suitable cryoprotectant medium such as in Todd Hewitt Glycerol broth or STGGB.

#### Point-of-Care Tests

Point-of-care tests such as NAATs and RADTs are commercially available for oropharyngeal specimens. When locally validated, they are acceptable alternatives to culture in clinical practice due to their ease of use and ability to produce results rapidly. Point-of-care tests are benchmarked against bacterial culture to report sensitivity (percentage of culture-positive specimens that are detected by the point-of-care test) and specificity (percentage of culture-negative specimens that are also negative by the point-of care test). Sensitivity and specificity of point-of-care tests will be different depending on the manufacturer and should be taken into consideration when selecting tests for surveillance.

#### Nucleic Acid Amplification Tests

Nucleic acid amplification tests are a molecular test for detecting Strep A deoxyribonucleic acid in pharyngeal swab specimens. The NAATs are available in rapid (<15 minutes to 1 hour) and easy-to-use commercial formats. Recent studies have found that NAATs have equal or greater specificity than most RADTs and are more sensitive (97.5%) point-of-care tests [17, 18]; thus, negative results do not require backup culture [19–21].

#### Rapid Antigen Detection Tests

Rapid antigen detection tests are used to identify the specific Strep A cell-wall antigen, the Lancefield group A carbohydrate. Depending on the manufacturer, these tests can be a latex agglutination assay, enzyme immunoassay, or optical enzyme immunoassay [22]. The RADTs provide rapid results (<10 minutes) and are an important, low-cost option for surveillance studies in resource-poor settings or locations where microbiology laboratories are not available [23, 24]. It is recommended that a throat culture or molecular test be performed for Strep A in children and adolescents if the RADT is negative and clinical presentation suggests Strep A pharyngitis due to their sensitivity (<90%) [25]. Current US pharyngitis “clinical” guidelines state that a positive RADT does not require a backup culture to initiate antibiotic therapy due to its high specificity [26]. However, when the objective is to conduct “surveillance” for *emm* types or antibiotic resistance among Strep A isolates, pharyngitis surveillance programs can perform culture on a representative sample of positive RADT specimens to obtain isolates for further characterization such as *emm* typing, antibiotic susceptibility testing, and whole-genome sequencing.

## SPECIMEN COLLECTION FOR BACTERIAL THROAT CULTURE

### Equipment and Supplies

The following equipment and supplies are needed: (1) gloves (need not be sterile); (2) sterile swabs (calcium alginate, rayon, Dacron, or nylon materials) [27]; (3) culture medium (eg, STGGB or room temperature stable alternative); (4) tongue depressor; (5) flashlight; (6) biohazard plastic bags, or clean plastic bags that can be labeled; (7) transport container; and (8) cooling bricks (if refrigerated storage is recommend for choice of culture medium).

### Methods of Sample Collection

Proper technique increases the yield of throat cultures in children. Persons collecting throat swabs should receive training in the following technique:

1. Verify the participant’s identity and label a sterile culture swab with the information requested by the protocol. This is typically 2 patient identifiers (e.g., initials and surveillance visit number), date, and surveillance or site identity.
2. Put on gloves.
3. Position the child to face the brightest part of the room. If available, have 1 person steady the child’s head.
4. Shine a bright flashlight or penlight in the child’s mouth.
5. Use the other hand to remove the throat swab from its protective covering taking care to keep the tip sterile.
6. Ask the participant to open the mouth widely, protrude the tongue and say, “ahhh” or pant to elevate the uvula. Swabbing is best done under direct visualization and with the aid of a tongue depressor placed approximately three quarters of the way to the posterior edge of the tongue to push the tongue down (inferiorly) firmly.
7. Rub the swab quickly but thoroughly over both tonsils (or tonsillar fossa) and the posterior pharyngeal wall of the pharynx using light pressure. Any exudate present should be swabbed. Other areas of the oral pharynx and mouth (eg, inside of cheeks) are not acceptable sites. Avoid contamination of the swab with saliva, the tongue, or oral cavity.
8. Carefully store swab in culture medium if culture is not performed immediately (ie, place swabs in STGGB medium [28] and keep the swabs cold until freezing or plating).

### Storage and Handling

The following storage and handling should be taken. (1) Make sure the top is screwed on or pushed on firmly in place. (2) All specimens should be stored in sealed biohazard plastic bags or inside a biohazard labeled sealed container. Store at the temperature required by culture medium. For example, room temperature storage is suitable for eSwabs (Copan, Italy), whereas refrigerated (in fridge) conditions are recommended for specimens stored in STGGB. (3) Sample collection documentation must be kept with specimens, but not in the same compartment in case of leakage.

### Documentation

The following documentation procedures should be taken. (1) Label all specimens: follow instructions on sticky label on tube/swab container; minimum information needed, eg, unique participant identification (ID) number, date specimen collected, and exactly what the specimen is (eg, blood, wound swab). (2) A specimen transport log form should be used, consisting of the following: place, date, and time of collection shipment; and contents of shipment including participant ID numbers, specimen types, and order of storage.

### Specimen Transfer

The following procedures should be taken for specimen transfer. (1) Place absorbent material in sealed biohazard bags with specimens in case of sample leakage. (2) Put into recommended portable transport container. For samples collected into storage medium with refrigeration recommended (ie, STGGB), store sealed bags in between ice cooler bricks. (3) Seal lid of portable container as instructed or with waterproof tape. (4) Label all containers clearly with the following: place, date, time of packing, and destination; and biohazard sticker, and if there is no sticker, write it in big letters using black marker. (5) Make sure the courier knows what the contents are, so they will not be left in a hot place and will be promptly delivered to the laboratory.

### Electrocardiogram

An age-adjusted (see Supplementary Appendix 4) prolonged PR interval is a minor manifestation of carditis. This should be performed at initial assessment and, if the PR interval is prolonged, then repeated several weeks/months later to confirm resolution. On occasion, a higher degree of heart block or other arrhythmias are seen.

## TYPES OF SURVEILLANCE RECOMMENDED

The selection of surveillance strategies depends on specific epidemiologic and clinical characteristics of the disease outcome of interest, the overall surveillance objectives, surveillance location, services accessibility, and the resources available to conduct surveillance (see Supplementary Appendix 5 for key surveillance definitions). For example, in resource-poor settings, the resources required for active surveillance and laboratory confirmation may not be available, and case-finding activities may be inhibited. Given that those in resource-poor settings are often most at risk for Strep A sequelae including ARF, surveillance is an important component of disease monitoring and control. Reliable burden estimates will inform the public health response to pharyngitis, advocate for vaccine use, and enable monitoring of the effect of interventions. Minimal and enhanced surveillance strategies for ARF are described in Table 3 to provide guidance for those with limited resources and those with greater capacity, respectively.

**Table 3.** Strategies for Surveillance of Acute Rheumatic Fever

*Minimal surveillance*
Minimal surveillance for ARF includes passive surveillance of primary healthcare facilities. Based on clinical signs and symptoms or a diagnosis recorded in health facility databases, or microbiological data from laboratory databases. Settings include primary healthcare clinics such as outpatient clinics, doctors’ offices and hospitals, and clinical laboratories. Participants are those who present to healthcare or other relevant settings, on their own accord. If the provider or surveillance officer determines that the case definition for ARF has been met, it can be recorded in electronic medical records, or a report provided to the surveillance system or local public health authorities. In the absence of access to microbiologic tests, diagnosis may be considered “possible” per the case definitions above. It is expected that the surveillance staff implementing surveillance have been appropriately trained identify the signs of ARF and use the Revised Jones Criteria appropriately. Standard case report forms may be provided to the health facilities or laboratories for completion and submission to the surveillance program. Appropriate when a minimum estimate of disease burden is considered adequate for surveillance purposes and the population at risk is well characterized demographically [29].
*Enhanced surveillance*
Enhanced surveillance of ARF includes prospective active case finding and laboratory confirmation among a large and well defined population. It requires timely detection of new cases to ensure appropriate testing is conducted—including confirmation of Strep A with ASO/ADB serology, throat culture or RADT/NAAT, and echocardiography to look for carditis at the time of symptomatic disease Participants are followed prospectively, ideally with frequent, regular contact, for a defined period using standard methods to collect demographic, clinical information, and repeat ASO/ADB titers to confirm rise 12–28 days later, if collected at symptom presentation. Repeat echocardiography should be performed for persons following symptom resolution and normalization of ESR/CRP to look for RHD. Well defined clinical practices and laboratory methods are established before surveillance and remain constant throughout the surveillance period, including policies for administering secondary prophylaxis and clinical follow-up of individuals with a definite or possible ARF diagnosis. Audits should be performed biannually to assess the completeness of case ascertainment, accuracy, timeliness, and laboratory performance. Regular feedback of data/information is provided to healthcare workers and others involved in the surveillance process. This critical communication engages healthcare workers in the process and informs their clinical practice.

A quality management plan should be written before the start of surveillance to establish and ensure the quality of processes, data, and documentation associated with surveillance activities. Furthermore, all surveillance should be conducted in accordance with ethical guidelines (see Supplementary Appendix 6).

## CASE ASCERTAINMENT AND OTHER SURVEILLANCE METHODOLOGY

For each data source, surveillance staff should adhere to the following (1) know the purpose of the data source; whether data have been routinely collected as part of patient care, mandatory collection of data under legal mandates, collected for research purposes, or other; (2) identify any legal mandates governing the operations of the data source that may affect the accessibility or quality of data from that source; and (3) describe the representative population for the data. Case ascertainment may be active or passive (see Supplementary Appendix 7).

The method of case ascertainment is an important part of surveillance and may significantly affect the accuracy and completeness of disease burden estimates.

### Surveillance Settings

#### Community

Only 1 prospective study on active case finding for ARF at the community level from a low- and middle-income country has been reported. The results revealed a much higher incidence of ARF among the population under surveillance than previously reported in the literature and provide a gold standard model for active case-finding efforts [30]. The resource-intensive model is appropriate for small, well defined populations (eg, demographic surveillance systems) rather than routine surveillance. The model combines community sensitization through messaging about signs of ARF and healthcare provider education to raise awareness of the clinical presentation of ARF. Messaging the population of interest can occur in many different forms, including radio, television, billboards/posters, word of mouth through community leaders, and direct school-based education programs. Discussion with local community and healthcare leaders before engaging in active case finding is important to determine which mode(s) of messaging would reach the most people in the population of interest and be most appropriate and culturally accepted. All messaging campaigns should convey the following to the community: (1) any child with fever and joint pain should present for medical evaluation for ARF; (2) any child with sudden-onset abnormal movements should present for medical evaluation; (3) any child with a common illness in the population of interest (eg, malaria and dengue) that may present similarly to ARF warrants medical evaluation; (4) instructions and contact information for the hospital(s) involved in the active case-finding surveillance where children can undergo evaluation.

#### Primary Healthcare

Primary healthcare settings can be used for active and passive surveillance. Active surveillance could recruit participants registered at the healthcare facility, requesting them to present to the clinic upon developing symptoms of ARF, including fever and joint complaints or involuntary movements. The surveillance staff would regularly reach out to families during the surveillance period (weekly) to facilitate presentation to primary healthcare clinics for laboratory testing and echocardiogram if symptoms develop. Primary healthcare surveillance is costly, resource intensive, and relies on engagement from surveillance staff, primary practitioners, and healthcare workers to maintain adequate retention rates, particularly for prospective longitudinal surveillance. Healthcare provider education on ARF presentation should be at the forefront of active surveillance efforts, especially in developing countries, because healthcare-provider knowledge of the link between streptococcal infection and ARF and preparedness to recognize and manage patients with ARF remains suboptimal [31]. All new healthcare providers joining the facility should be trained and the time and content of the training formally documented.

Active surveillance staff in hospitals and healthcare centers should establish multiple levels of case ascertainment, including the following: (1) regular liaison with hospital medical staff in pediatrics and pediatric surgery, and routine review of echocardiography and streptococcal serology results; and (2) routine review of all admissions with an overinclusive list of admission diagnoses (due to the frequent misdiagnosis of ARF). Surveillance staff should be aware that ARF cases do not always present to general pediatric or medical services; surgeons (usually orthopedic), neurologists, and cardiologists often look after ARF cases when first admitted to hospital.

Passive surveillance in primary care settings involves recording data on patients who present to primary healthcare clinics. Although often limited in diagnostics, primary care centers can play a pivotal role by contributing data on adverse outcomes and case fatalities as they manage patients in the outpatient setting after diagnosis. Electronic medical records can assist surveillance, allowing data extraction at regular intervals. Therefore, we recommend that surveillance systems incorporate passive surveillance through medical record data as described in Supplementary Appendix 8.

### Participant Eligibility

A surveillance protocol should clearly describe enrollment eligibility criteria. Most protocols would benefit from surveying children aged from 5 to 15 years; however, age eligibility can vary between sites, depending on local needs and capacity. Children already receiving prophylactic antibiotics for any cause (eg, RHD, surgical procedures, human immunodeficiency virus, sickle cell, etc) should not be excluded from ARF surveillance; however, the use of prophylactic antibiotics should be recorded. Unless specifically relevant to the surveillance aims, we also recommend including persons with underlying immunocompromising conditions or chronic diseases in ARF surveillance. In vaccine efficacy trials, persons who are immunocompromised or on prophylactic antibiotics should be excluded from phase 1 and phase 2 trials because it may be difficult to interpret serologic data and culture results, which impact vaccine efficacy measurements.

The surveillance population includes all eligible at-risk people from which cases of ARF are identified. This population, or denominator, must be well characterized *a priori* to derive meaningful disease burden estimates. Without an accurate account of all people in the population who could potentially be evaluated for ARF, disease estimates may be under- or overestimated [32, 33].

Some settings allow population-wide data on disease burden to be recorded and analyzed. Examples include household surveillance in a representative sample in a community or healthcare setting that serves the entire community. In these cases, the surveillance population would be defined as all eligible people who reside in the community. Data accuracy must be assured if government-derived census data are used to determine the community’s demographic profile, such as the number of people in relevant age categories. Ongoing, multiyear surveillance might be necessary to generate reliable burden estimates if surveillance extends over a long period of time or if the population is not stable because of mobility or other logistic factors.

In instances where select primary healthcare facilities serve a portion of a population residing in the geographical catchment area, healthcare utilization surveys can be used to estimate the denominator corresponding to the cases of interest, improving the accuracy of disease burden estimates and enabling rate calculations [34]. The denominator is the number of patients within the geographical catchment area who would be expected to attend that primary healthcare facility if signs and symptoms of ARF develop. Cases not residing in the defined catchment area should be excluded. In an ideal setting, the denominator population should be defined before surveillance begins.

When undertaking surveillance in a sample of schools and/or classrooms, the surveillance population is the number of children who agree, and have parental or guardian appropriate consent, to participate in surveillance. The results can be generalized to the entire community if schools and classes are randomized at the start of surveillance or appropriate demographic characteristics of participants can be weighed against the characteristics of the catchment population.

## ADDITIONAL SURVEILLANCE CONSIDERATIONS

### Administrative Database Review

Codes used to identify ARF in electronic medical records are shown in Table 4.

**Table 4.** EMR-Specific Codes for ARF

**For Primary Healthcare Systems, ARF Is Coded Under:**	
*International Classification of Primary Care, version 2 (ICPC-2) system*	K 71 (Rheumatic fever/heart disease)
**For Hospital Data Systems, ARF Is Coded Under:**	
*International Statistical Classification of Diseases and Related Health Problems, Tenth Revision (ICD-10)*	I00 (Rheumatic fever without heart involvement)
	I01 (Rheumatic fever with heart involvement)
	I02 (Rheumatic chorea)

Abbreviations: ARF, acute rheumatic fever; EMR, electronic medical record.

### Implications for Secondary Prophylaxis and Follow-up

A complete understanding of local healthcare system infrastructure is vital to guarantee the availability of necessary administration supplies and trained healthcare workers for administering benzathine penicillin G (BPG). Those responsible for conducting surveillance should remain alert to the possibility that harm can be caused to both individuals and communities. Those conducting surveillance have an obligation to identify potential harm beforehand, to monitor for harm during and after surveillance, and to put in place processes to mitigate harm. An example of harm laid out in the WHO Guidelines on Ethical Issues outlines “inadequate treatment” as a source of physical harm. Those conducting surveillance can mitigate this harm by ensuring that intramuscular BPG is available for monthly prophylaxis to those diagnosed with ARF.

Surveillance staff should ensure a predetermined policy exists that adheres to local guidelines (or World Health Organization [WHO] if no local guidelines are available) for administering secondary prophylaxis and clinical follow-up of individuals with a definite or possible ARF diagnosis. Possible ARF generally requires a similar treatment approach as definite ARF, depending on local definitions of possible and/or probable ARF. Recommended management varies according to local considerations and available resources.

### Registers For Acute Rheumatic Fever

Joint ARF/RHD registers have a central role in supporting prophylaxis delivery, facilitating ongoing care delivery for people living with ARF, and program evaluation. They can also be used for research, managing surgical waiting lists, and providing focused education support to people with a history of ARF or living with RHD. The registers also provide important natural history data for ARF and RHD. Surveillance staff pursuing active case finding for ARF should establish a register for those diagnosed in screening programs to facilitate follow-up, administration of secondary prophylaxis, and contribute to natural history data.

### Surveillance Period

Defining the surveillance duration depends on the availability of resources to support the surveillance system and the time needed to achieve the surveillance objectives. A minimum of 1 year is recommended due to the influence of seasonality (see below). Multiple years of surveillance are generally required to evaluate temporal trends, M or *emm* type distribution, or the impact of an intervention such as introducing a vaccine program.

### Season

If possible, surveillance staff should conduct surveillance across all seasons to capture the changes in disease occurrence over time. In areas where seasonality is well described, limiting surveillance to months when most cases are likely to occur has efficiencies but will inflate incidence estimates and should not be used to extrapolate to annual incidence rates. Continuous surveillance is optimal but may not be possible due to school holidays, extended absences from school to tend to farms or other family or community duties, access to remote areas during wet seasons, and closure of communities for cultural reasons.

### Community Engagement/Involvement

Community engagement helps provide a considered approach to surveillance and ensures that the project has community value. It also ensures that the community has an opportunity to clearly express their values and concerns and develop a degree of ownership. The time required to forge relationships between surveillance staff and communities should not be underestimated and must be built into the surveillance protocol at the outset.

The level of community involvement in the design, implementation, monitoring, and evaluation of surveillance will depend on available resources and community capacity. Key stakeholders include community leaders, teachers, health staff, and volunteers.

### Terms to Describe Disease Burden

The burden of ARF can be described in terms of incidence as either cumulative incidence or incidence rate. Both measures have new episodes of ARF occurring in the observation period as their numerators, although first-ever and recurrence episodes should be differentiated, with the sum of these measures providing total incidence. An episode is classified as a recurrence when a person experiences a new ARF episode, by convention defined as being >90 days since their previous episode. The denominators for both cumulative incidence and incidence rates capture population size but treat the time at risk differently.

*Cumulative Incidence*. For cumulative incidence, the denominator is the total number of people at risk of developing the disease during a defined period. Thus, the denominator is the number of people in the study population who are episode free at the beginning of the study period. This does not account for the time that people are not at risk or recurrent episodes in the same person.

*Incidence Rates*. For incidence rates, the denominator is the total time that all persons in the population were followed up (often called person-time), even when their dates of entry and departure from the population/group were different. Incidence rates that use exact person-time may not be that practical in many settings. Thus, for population-based incidence rates, the average population during the time interval can be used as a proxy for summed person-years of observation. In most settings, the denominator will simply be an estimate of the population size (from a census) multiplied by the time period of observation (eg, years). We recommend that incidence rates are expressed in terms of person-time rather than cumulative incidence because it is easier to interpret and compare between settings. Incidence rates are also useful when individuals are actively followed up accurately over time such as in active surveillance as they account for recurrent episodes (and potentially progression to RHD) in the same individual, providing useful information about the course of the disease and effectiveness of secondary prophylaxis. Research studies and disease registers might provide data of sufficient quality to provide exact estimates of person-time as the denominators.

## DATA COLLECTION AND CASE REPORT FORMS

Case report forms should be used to collect only the information required to achieve the surveillance objectives. See Supplementary Appendix 9 for a list of recommended and optional variables for inclusion in all case report forms. Case report forms can be paper based but secure electronic data forms are increasingly used. Electronic case report forms offer a number of benefits such as early detection of cases and timely information flow, relatively inexpensive to operate, and improved data quality (accuracy and data completeness) via imbedded validation checks.

### Consent

Before initiating an assessment and collecting data or specimens, consent for participation in the surveillance program may need to be obtained based on the determination of an institutional review board. For children, consent needs to be obtained from their parent or legal guardian, and before examining, request permission (assent) from the child. Consent should be voluntary and based on sufficient information and an adequate understanding of the proposed surveillance program and the implications of participation. Flip charts and interpreters may help improve information delivery so that participants are clear about to what they are consenting. If consent is not obtained, do not proceed. For prospective active surveillance programs, each participant must be informed that participation in the project is voluntary and that they are free to withdraw, without justification, from the surveillance system at any time without consequences. Note that the age at which consent can and should be given by the child will vary between countries and/or jurisdictions. It is the responsibility of surveillance staff to confirm the requirements of local, regional, or national authorities. Informed consent may be obtained for surveillance/throat examination, photos of throat, administration of throat swabs, and storage of swabs for future use such as genetic sequencing, transcriptome analysis.

Improving the global burden estimate through disease surveillance is critical to further characterize the epidemiology of ARF and inform future vaccine implementation strategies. The type of surveillance conducted depends on the population and resources as discussed above. General surveillance information includes a unique identifier, date and time of enrollment, and site where participant is seen such as setting, location, postcode, state/province/region, and country. Each encounter should also record a surveillance visit number/echocardiogram number if multiple echocardiograms are performed. Moreover, key demographic information includes date of birth or age in years (if date of birth not available), sex, ethnic origin/race, residential postcode, state, and country. Finally, clinical and epidemiologic information includes signs and symptoms including those listed in the 2015 Jones Criteria, epidemiologic risk factors, echocardiogram results, and details of prescribed antibiotic prophylaxis and adherence.

## Supplementary Data

Supplementary materials are available at *Open Forum Infectious Diseases* online. Consisting of data provided by the authors to benefit the reader, the posted materials are not copyedited and are the sole responsibility of the authors, so questions or comments should be addressed to the corresponding author.

## Supplementary Material

ofac252_Supplementary_Data
