# Supplementary material for: Standardization of Epidemiological Surveillance of Acute Rheumatic Fever
Source: Open Forum Infect Dis. 2022 Sep 15;9(Suppl 1):S41–9. doi: 10.1093/ofid/ofac252 (PMC9474936; doi:10.1093/ofid/ofac252)
Supplement: ofac252_Supplementary_Data [file ofac252_supplementary_data.docx]

**Standardization of Epidemiological Surveillance of Acute Rheumatic Fever**

Supplementary Appendices

Table of Contents

[Appendix 1: Important Considerations when Applying the 2015 Revised Jones Criteria 2](#_Toc112339730)

[Appendix 2: Comparison of Tests Used to Confirm Preceding Strep A Infection for ARF 3](#_Toc112339731)

[Appendix 3: Upper Limit of Normal (80th centile) Values for Serum Streptococcal Antibody Titers in Children and Adults in Tropical Settings Where Strep A is Endemic 5](#_Toc112339732)

[Appendix 4: Upper Limits of the Normal PR interval; Age Adjusted 5](#_Toc112339733)

[Appendix 5: Definitions of Key Surveillance Terms 6](#_Toc112339734)

[Appendix 6: Good Practice and Ethical Considerations 7](#_Toc112339735)

[Appendix 7: Comparisons of Advantages and Disadvantages of Active and Passive Surveillance 9](#_Toc112339736)

[Appendix 8: Administrative Health Databases 10](#_Toc112339737)

[Appendix 9: Variables for Inclusion in Acute Rheumatic Fever (ARF) Datasets 11](#_Toc112339738)

## Appendix 1: Important Considerations when Applying the 2015 Revised Jones Criteria

| ^a^ Low-risk populations are those with ARF incidence ≤2 per 100,000 school-aged children or all-age rheumatic heart disease prevalence of ≤1 per 1,000 population per year. |
| --- |
| ^b^The standard used to define pathological mitral regurgitation (MR) and aortic regurgitation (AR) associated with chronic RHD (RHD Chapter, Appendix 1) is the same as for MR and AR seen in ARF. Should there be any doubt regarding the etiology of MR or AR on echocardiography, in addition to morphological changes, the following features support a diagnosis of acute carditis:   - Pathological regurgitation involving mitral and aortic valves - Posteriorly directed mitral regurgitant jet, because the most common mechanism for MR in acute carditis is prolapse of the anterior mitral valve leaflet - Evolution of valvulitis over weeks (increasing or decreasing in severity).   Pathological regurgitation should always be graded as mild, moderate, or severe using continuous-wave and color-Doppler. |
| ^c^ Polyarthralgia should only be considered a major manifestation in moderate-to-high-risk populations after exclusion of other causes. As in past versions of the criteria, erythema marginatum and subcutaneous nodules are rarely “stand-alone” major criteria. Additionally, joint manifestations can only be considered in major or minor categories, not both, in the same patient. |
| ^d^ CRP value must be greater than upper limit of normal for laboratory. Furthermore, peak ESR values should be used because ESR can evolve during ARF. |

Abbreviations: AR, aortic regurgitation; ARF, acute rheumatic fever; CRP, C-reactive protein; ESR, erythrocyte sedimentation rate; MR, mitral regurgitation; RHD, rheumatic heart disease.

Adapted from: Dougherty S, Nascimento B, Carapetis J. Chapter 3 - Clinical Evaluation and Diagnosis of Acute Rheumatic Fever. In: Dougherty S, Carapetis J, Zühlke L, Wilson N, eds. *Acute Rheumatic Fever and Rheumatic Heart Disease.* San Diego (CA): Elsevier; 2021:31-54.

## Appendix 2: Comparison of Tests Used to Confirm Preceding Strep A Infection for ARF

| **Gold Standard Approach** | **Alternative Approach** | **Limitations** |
| --- | --- | --- |
| **Elevated ASO or ADB Titer(s)** | |  |
| - If patient presents within two weeks of illness, collect lab values on more than one occasion separated by at least 14 days (4-6 weeks preferred) to demonstrate rising titers: a two-fold increase in titer from acute to convalescence is considered acceptable, however a four-fold increase is the gold standard - *ASO titers typically peak 1–3 weeks after infection; if presentation is >3 weeks, obtain ASO titer at presentation. Repeating for a second time is of little utility - Develop age-stratified ULN values for serum ASO and ADB titers in a subset of individuals without a recent streptococcal infection in the surveillance population of interest | - A single value above the ULN is considered sufficient for a preceding Strep A infection if not feasible to obtain multiple specimens. This is the most common approach, especially because the first sample is often delayed due to late presentation, or a long latent period between infection and onset of ARF may mean that antibody titers have already begun to rise at the time of presentation - See Appendix 3 for ASO and ADB ULN titers when local derivations are not available | - Development of local population ULN is not always possible due to logistics, costs, or surveillance being conducted in areas with high prevalence of streptococcal infections and thus difficulty finding children without recent infection |
| **Positive Throat Culture for Group A β-hemolytic Streptococci** | | |
| - Specimens should be obtained from the surface of both tonsils and the posterior pharyngeal wall - The sample should be cultured on a sheep-blood agar plate and incubated at 35–37°C for 18–24 h before reading. If no growth occurs, Incubate for an additional 24 h before concluding that the culture is negative | - NAAT assays have been studied extensively and are highly sensitive for detecting Strep A.^1^ - NAAT assays offer a quick and reliable alternative to bacterial culture | - False negatives may occur if child is treated with antibiotics prior to culture collection - Strep A can exist in the carrier state; a positive throat culture does not necessarily confirm active infection - Patients with ARF typically present weeks after initial Strep A infection |
| **Positive Rapid Diagnostic Test** | | |
| - Specimens should be obtained from the surface of both tonsils and the posterior pharyngeal wall - Use NAAT due to higher sensitivity than traditional RADT | - Use of RADT when NAAT test is not available - Validation of RADT to be used in population with culture confirmation prior to use when possible | - Strep A can exist in the carrier state; a positive throat culture does not necessarily confirm active infection - Patients with ARF typically present weeks after initial Strep A infection - NAAT equipment is costly and does not differentiate between viable and nonviable bacteria in the specimen - RADT tests have varying sensitivities that do not differentiate between viable and nonviable bacteria, and negative results require culture |
| **Group A β-hemolytic streptococci from Active Impetigo Lesion** | | |
| - Perform full body skin exam to locate most active lesions - Sample active lesion, selecting the most purulent lesion (see Appendix 9 for further guidance) | - Sample crusted lesions if no purulent lesions present | - Older lesions are more likely to become co-infected with *Staphylococcus aureus* or not grow on culture |

Abbreviations: ARF, acute rheumatic fever; ASO, antistreptolysin O; ADB, anti-DNase B; lab, laboratory; NAAT, nucleic amplification test; RADT, rapid antigen detection test; Strep A, Streptococcus pyogenes; ULN, upper limit of normal.

Adapted from: Parker KG, Gandra S, Matushek S, Beavis KG, Tesic V, Charnot-Katsikas A. Comparison of 3 Nucleic Acid Amplification Tests and a Rapid Antigen Test with Culture for the Detection of Group A Streptococci from Throat Swabs. *J Appl Lab Med.* 2019;4(2):164-169.

## Appendix 3: Upper Limit of Normal (80th centile) Values for Serum Streptococcal Antibody Titers in Children and Adults in Tropical Settings Where Strep A is Endemic

| Age Group (years) | Upper Limit of Normal (international units/mL) | |
| --- | --- | --- |
|  | ASO Titer | ADB Titer |
| 1–4 | 170 | 366 |
| 5–14 | 276 | 499 |
| 15–24 | 238 | 473 |
| 25–34 | 177 | 390 |
| >35 | 127 | 265 |

Abbreviations: ASO, antistreptolysin O; ADB, anti-DNase B

From: Steer AC, Vidmar S, Ritika R, et al. Normal ranges of streptococcal antibody titers are similar whether streptococci are endemic to the setting or not. *Clin Vaccine Immunol.* 2009;16(2):172-175.

## Appendix 4: Upper Limits of the Normal PR interval; Age Adjusted

| **Age Group (years)** | **Duration (seconds)** |
| --- | --- |
| 3-12 | 0.16 |
| 12-16 | 0.18 |
| ≥17 | 0.20 |

## Appendix 5: Definitions of Key Surveillance Terms

| **Syndromic surveillance** | Syndromic surveillance refers to the use of a clinical syndrome – a constellation of symptoms and signs – as the case definition for detection of suspect cases. Syndromic surveillance can be used for initial case detection, but laboratory confirmation should occur to increase the accuracy of the system^1^. |
| --- | --- |
| **Active surveillance** | Active case detection means that designated public health surveillance staff actively detect cases and report to the public health system ^1^. |
| **Passive surveillance** | Passive case detection means that health facility staff detect and report cases to the public health system^1^ |
| **Facility-based surveillance** | Facility-based surveillance is based on ascertainment of cases in persons who seek care at health facilities, including outpatient clinics, doctors’ offices, hospitals and emergency departments ^1^. |
| **Sentinel-site surveillance** | Sentinel-site surveillance refers to a system that captures cases at one or more specialized sites, such as hospitals, clinics, schools or pharmacies^1^. |
| **Community-based surveillance** | Community-based surveillance is the systematic detection and reporting of events of public health significance within a community-by-community members. Community-based surveillance enables earlier detection of the disease of interest and captures illnesses in persons who do not seek care in a hospital^2^ |
| **Population-based surveillance** | Population-based surveillance attempts to capture all cases in a well-defined catchment population (for example, the entire population of a country). |
| **Healthcare utilization surveys** | Healthcare utilization surveys characterize the health care-seeking behavior of ill persons by describing where ill persons sought health care for their illnesses, and soliciting reasons for not seeking health care ^3^ |
| **Unique identifier** | Unique identifiers are unique numbers or numbers and letter combinations that are allocated to a specific individual person. |

^1^World Health Organization. Surveillance standards for vaccine-preventable diseases. 2018.

^2^World Health Organization. A definition for community-based surveillance and a way forward: results of the who global technical meeting, france, 26 to 28 june 2018. *Eurosurveillance.* 2019;24(2).

^3^Deutscher M, Van Beneden C, Burton D, et al. Putting surveillance data into context: the role of health care utilization surveys in understanding population burden of pneumonia in developing countries. *Journal of epidemiology and global health.* 2012;2(2):73-81.

## Appendix 6: Good Practice and Ethical Considerations

**Monitoring/Audit**

A systematic and independent audit of surveillance systems should be undertaken to ensure that surveillance and surveillance-related activities were conducted following the relevant surveillance protocol, standard operating procedure, ethical guidelines, and regulatory requirement(s) established by local public health. Existing surveillance review tools can be modified to guide the investigation (e.g., WHO’s ‘[Tools for a surveillance review: Vaccine Preventable Diseases Surveillance Standards](https://www.who.int/publications/m/item/vaccine-preventable-diseases-surveillance-standards-annex1)’). Surveillance as part of a clinical study should adhere to the ICH Guidelines for Good Clinical Practice^1^.

**Quality Control and Quality Assurance**

A quality management plan should be written before the start of surveillance to establish and ensure the quality of processes, data, and documentation associated with surveillance activities. It encompasses both quality control (QC) and quality assurance (QA) activities.

Surveillance systems should develop a SOP to ensure confidentiality for all cases, ensure that clinical specimens and bacterial isolates obtained are not compromised by human and processing errors, validate data integrity, and maintain multiple layers of security. A SOP will ideally detail:

- Data storage. Including participants’ unique surveillance ID numbers in each respective dataset enables linkage to other datasets, such as hospital admissions, facilitating the capture of complications and ensuring that all personal identifying information is removed from research/surveillance datasets.
- Data evaluation for protocol compliance and source document accuracy.
- Document review (e.g., specimen tracking logs, questionnaires), who is responsible, and frequency.
- Who the responsible person is for addressing QA issues (correcting procedures that do not comply with the surveillance protocol) and QC issues (correcting errors in data entry).
- Staff training activities and processes for documenting surveillance staff training.
- Maintenance and strict adherence to surveillance delegation log (list of staff involved in the surveillance and their duties/roles).
- Clinical and laboratory SOP and accreditation.
- Regular audits of surveillance data to ensure accuracy and completion.
- System for periodic and refresher training for surveillance team.

**Ethics of Surveillance**

The global network of WHO Collaborating Centres for Bioethics in collaboration with the U.S. Centers for Disease Control and Prevention developed ethical guidelines for public health surveillance, including common good, respect for persons, and good governance. The guidelines cover the (i) broad responsibility for undertaking surveillance and subjecting it to ethical scrutiny; (ii) obligation for ensuring appropriate protection and rights; (iii) considerations in making decisions about how to communicate and share surveillance data. The guidelines are available at <https://apps.who.int>. Countries should implement these guidelines and monitor them regularly. As appropriate, surveillance protocols should adhere to existing country-specific ethical guidelines.

^1^U.S. Department of Health and Human Services. *E6(R2) Good Clinical Practice: Integrated Addendum to ICH E6(R1) Guidance for Industry* Maryland 2018.

## Appendix 7: Comparisons of Advantages and Disadvantages of Active and Passive Surveillance

| **Advantages** | **Disadvantages** |
| --- | --- |
| ***Active surveillance*** |  |
| - Sensitive system that facilitates early detection of new cases, contributing to prevention of post-infection sequalae - Higher case ascertainment rate - More accurate identification of cases - Ability to verify information in the case of missing data or suspected data entry errors - Data collected can be comprehensive and specific to the surveillance objectives - Can evaluate the quality and effectiveness of case-finding process, thus minimizing selection bias - Allows real-time analysis and ability to respond/modify approach to surveillance and care - Can promote disease awareness and good health practices | - Can be costly and resource-intensive - Requires dedicated surveillance staff and/or extensive training and upskilling - Can be demanding on surveillance sites - Barriers to accessing communities (e.g., distance/cultural barriers) |
| ***Passive surveillance*** |  |
| - Can be conducted retrospectively - Requires fewer resources than active surveillance - Can support real-time reporting | - Responsibility for reporting new cases lies with the healthcare workers/laboratory staff; thus, it can be difficult to ensure consistency of reporting by healthcare providers - Difficulties caused by lack of standardization in terms of case definitions and coding - Tends to under-report disease - Often difficult to confirm data recording or entry errors retrospectively - Commonly associated with incompleteness of data recording or of microbiological studies - Vulnerable to bias due to differences in physicians’ inclination to perform microbiological confirmation |

## Appendix 8: Administrative Health Databases

Administrative data from laboratory datasets and electronic medical records (EMRs) from primary healthcare and emergency departments covering whole communities can provide a timely and cost-effective surveillance option.

An important consideration when using EMRs to calculate disease estimates in a population is that the data are collected and coded as part of service delivery rather than for surveillance purposes. As such, EMRs are often prone to missing data on key fields and require the conversion of unstructured/narrative text, which can be resource-intensive and subjective. For EMRs that include or rely on free text, new methods in machine learning or deep learning could improve case identification^1,2^. Further, data are limited to patients who attend health services, and are subject to variance in physician’s propensity to seek microbiological confirmation, which may be subject to bias (e.g., more severe infections, more clinically ambiguous, one not responding to treatment) and underestimate disease incidence. However, an advantage to administrative data is that, in well-established systems, data are collected systematically, well-structured and are often population-based. EMRs can form the basis of enhanced surveillance by using an additional data collection form to augment routinely collected data.

Routinely collected clinic data may be insufficient for evaluating potential cases against the full criteria required to meet surveillance case definitions, especially when microbiological testing is not routinely conducted or recorded. Further, the data may be insufficient for addressing other surveillance objectives, such as variant typing and antimicrobial susceptibility testing.

^1^Ayala Solares JR, Diletta Raimondi FE, Zhu Y, et al. Deep learning for electronic health records: A comparative review of multiple deep neural architectures. *J Biomed Inform.* 2020;101:103337.

^2^Wang S, Lengeler C, Mtasiwa D, et al. Rapid Urban Malaria Appraisal (RUMA) II: epidemiology of urban malaria in Dar es Salaam (Tanzania). *Malar J.* 2006;5.

## Appendix 9: Variables for Inclusion in Acute Rheumatic Fever (ARF) Datasets

Below are highly recommended and other suggested variables for inclusion in all case report forms. Where possible, the case report form should include a list of choices rather than an open text field for data capture.

| **Category of Variables** | **Required Variables** | **Optional Variables** |
| --- | --- | --- |
| General | - Unique ID number - Date of enrolment - Enrolment site |  |
| Demographics | - Age in years - Sex | - Race/ethnicity - Date of birth - Residential Address |
| Medical history | - Date of symptom onset (per symptom) - History and diagnosis date of:   - ARF: Definite, Possible, or No   - RHD: Yes, No, Unknown   - Prescribed BPG prophylaxis?: Yes, No | - Date of last ARF episode - Manifestations of ARF at prior episodes - Valves affected and severity of pre-existing RHD - Date of last dose of BPG or % missed oral prophylaxis in last month - Medication received prior to arriving at hospital:   - Anti-inflammatory:   - Paracetamol/acetaminophen   - Codeine   - Naproxen   - Other (specify)   - Antibiotic   - Benzathine penicillin G   - Oral penicillin   - Other (specify) |
| Classification and presentation of disease | - Diagnostic category: Definite Initial, Definite Recurrent, Possible Initial, Possible Recurrent - Major manifestations - Carditis - Arthritis: Mono/Polyarthritis - Polyarthralgia - Chorea - Erythema marginatum - Subcutaneous nodules - Minor manifestations - Monoarthralgia - Fever - Elevated ESR/CRP - Prolonged PR interval | - Carditis: Valve affected and severity   - MR – Mild/Mod/Severe   - AR – Mild/Mod/Severe   - MS – Mild/Mod/Severe   - AS – Mild/Mod/Severe   - Arthritis: Specific joint(s) affected   - Migratory: Yes, No   - Fever   - Peak Temperature   - ESR: Peak ESR   - CRP: Peak CRP   - PR interval: specify |
| Evidence of Strep A infection | - Throat swab for culture (positive/negative/not done) - Throat swab for rapid antigen (positive/negative/not done) - Skin swab for culture (positive/negative/not done) - ASO titer (date taken and titer, date and titer if repeated) - Anti-DNase B titer (date taken and titer, date and titer if repeated) - Has the patient had a recent sore throat (Y/N – if yes, date of onset) | - Has the patient had a recent sore throat (Y/N – if yes, date of onset) |
| Additional diagnostic testing performed |  | - Blood culture (Yes, No, Date, Result) - Joint aspiration (Yes, No, Date, Result) - Chest X-ray (Yes, No, Date, Result) - ANA (Yes, No, Date, Result) - Malaria (Yes, No, Date, Result) - Other serology (Specify, Date, Result) - Additional diagnosis given at presentation: specify |
| Treatment plan | - Antibiotics: Yes, No   - BPG/ oral penicillin / Other   - Date of first dose - Enrolled in ARF/RHD registry: Yes, No | - NSAIDS: Yes, No   - Aspirin/naproxen/other - Paracetamol: Yes, No - Corticosteroids: Yes, No - Heart Failure Meds: Yes, No - Chorea medications: Yes, No   - Carbamazepine/Valproic acid/Haloperidol/Other - Other meds: Yes, No |

Abbreviations: MR; mitral regurgitation, AR; aortic regurgitation, MS; mitral stenosis, AS; aortic stenosis, ESR; erythrocyte sedimentation rate, CRP; C-reactive protein, BPG; benzathine penicillin G, ANA; antinuclear antibodies
